# Molecular characteristics and successful management of a respiratory syncytial virus outbreak among pediatric patients with hemato-oncological disease

**DOI:** 10.1186/s13756-018-0316-2

**Published:** 2018-02-13

**Authors:** Claas Baier, Sibylle Haid, Andreas Beilken, Astrid Behnert, Martin Wetzke, Richard J. P. Brown, Corinna Schmitt, Ella Ebadi, Gesine Hansen, Thomas F. Schulz, Thomas Pietschmann, Franz-Christoph Bange

**Affiliations:** 1 Institute for Medical Microbiology and Hospital Epidemiology, Hannover Medical School, Carl-Neuberg-Straße 1, 30625 Hannover, Germany; 2 Institute for Experimental Virology; Twincore- Centre for Experimental and Clinical Infection Research; a joint venture of Hannover Medical School (MHH) and Helmholtz Centre for Infection Research (HZI), Hannover, Germany; 3 Department of Paediatric Haematology and Oncology, Hannover Medical School (MHH), Hannover, Germany; 4 Department for Paediatric Pneumology, Allergy and Neonatology, Hannover Medical School (MHH), Hannover, Germany; 5 Institute of Virology, Hannover Medical School (MHH), Hannover, Germany

**Keywords:** RSV, Respiratory syncytial virus, Outbreak, Infection control, Molecular typing, Pediatric patients, Hematology and oncology, Cancer patients

## Abstract

**Background:**

Respiratory syncytial virus (RSV) is responsible for upper and lower respiratory tract infection in adults and children. Especially immunocompromised patients are at high risk for a severe course of infection, and mortality is increased. Moreover RSV can spread in healthcare settings and can cause outbreaks. Herein we demonstrate the successful control and characteristics of a RSV outbreak that included 8 patients in our Department of Pediatric Hematology and Oncology.

**Methods:**

We performed an epidemiologic investigation and a molecular analysis of the outbreak strains. Moreover we present the outbreak control bundle and our concept for RSV screening in the winter season.

**Results:**

RSV A and B strains caused the outbreak. RSV B strains affected 3 patients, 2 of whom were co-infected with RSV A. Exactly this RSV A strain was detected in another 5 patients. Our multimodal infection control bundle including prophylactic RSV screening was able to rapidly stop the outbreak.

**Conclusion:**

An infection control bundle in RSV outbreaks should address all potential transmission pathways. In pediatric settings the restriction of social activities might have a temporal negative impact on quality of life but helps to limit transmission opportunities. Molecular analysis allows better understanding of RSV outbreaks and, if done in a timely manner, might be helpful for guidance of infection control measures.

## Background

Respiratory syncytial virus (RSV) of the family *Pneumoviridae* is a single stranded RNA-virus with two antigenic different subtypes (A and B). It causes upper and lower respiratory tract infection (URTI and LRTI) in children and adults in a seasonal pattern [1–4]. The median incubation period is 4.4 days [5], ranging from 2 to 8 days. Human to human transmission takes place via droplets as well as direct and indirect contact (e.g. contaminated surfaces or hands of medical staff). Patients with hemato-oncological disease are at risk for severe RSV-caused infection - especially in the context of hematopoietic stem cell transplantation (HSCT) [6, 7]. In the literature varying RSV-related case fatality rates are reported in children with cancer to range from 5% to 33% [8–10].

Respiratory tract infection (RTI) due to RSV is typically community/household-acquired. RSV is a member of the so called community-acquired respiratory viruses such as influenza virus. Nevertheless, hospital (nosocomial) acquisition is possible as well and transmission may occur by other infected patients, staff or visitors [11, 12]. RSV outbreaks in inpatient pediatric oncologic care facilities and in adult hematology and oncology units have been described [9, 12–16]. An understanding of transmission pathways helps to guide adequate outbreak control measures and to implement prophylactic measures. Finally, RSV-caused respiratory tract infections are a differential diagnosis worth considering in neutropenic cancer patients with fever [17, 18].

Therapeutic options for patients in hematopoietic stem cell transplantation and with intensive cancer therapy, who are severely infected by RSV, include the use of systemic or aerosolized ribavirin and polyclonal intravenous immunoglobulins (IVIG)[19, 20]. The RSV-specific monoclonal antibody Palivizumab has been used for treatment and for passive immunization (prophylaxis) in high risk pediatric patient groups [19–21].

Here we describe the successful management and characteristics of a RSV outbreak in a single non-HSCT pediatric hematology and oncology ward including 8 patients in March and April 2016. Moreover, we show the results of the molecular strain analysis.

## Methods

### Outbreak Case definition

An outbreak case is a patient with a positive RSV laboratory testing in samples from the upper or lower respiratory tract and a definite or possible nosocomial onset. A definite nosocomial case was defined as a positive RSV laboratory testing on day 5 or later of the hospital stay. A possible nosocomial case was defined as a positive RSV laboratory testing on day 2 to 4 of hospital stay. Patients who were admitted to the ward with a new positive RSV laboratory testing, and had been on the ward within 8 days prior to admission were also considered a possible nosocomial case. All patients that were accommodated in the same room with cases were considered contacts.

### Processing of patient specimens

A combined nose/throat swab was taken for routine viral diagnostics. Material from the lower respiratory tract was suitable as well.

Samples taken for diagnostic purposes were processed at the Institute of Virology using real-time RT-PCR or direct fluorescent antibody (DFA) staining. RNA was extracted from the specimens using a QiaAmp Viral RNA Mini Kit in a QIAcube according to the manufacturer’s instruction (Qiagen, Hilden, Germany). cDNA synthesis, amplification and detection of nucleic acid were performed in an Applied Biosystems® 7500 Real-Time PCR System (Life Technologies, Carlsbad, California) by a commercially available one-step real-time RT-PCR kit (RSV/hMPV r-gene® PCR Kit, bioMérieux, Nürtingen, Germany) according to the manufacturer’s instructions. For DFA staining a ready to use FITC (fluorescein isothiocyanate) -labeled monoclonal RSV antibody (LIGHT DIAGNOSTICS, Merck, Darmstadt, Germany) was used according to a protocol described before [22]. PCR and DFA did not differentiate between RSV A and B. One diagnostic specimen was tested using a point-of-care test (POCT) system (Sofia, Quidel, Kornwestheim, Germany), which is available in the pediatric emergency room.

### Strain typing

Nasopharyngeal aspirates from 6 outbreak patients (case 1, 2, 3, 5, 6, and 7) were taken only for strain typing purposes on one occasion (March18^th^), which were exclusively processed at the Institute for Experimental Virology, Twincore - Centre for Experimental and Clinical Infection Research. In addition, selected archived (frozen) samples taken for diagnostic purposes from outbreak patients (case 2, 3, 4, 5, 7, 8) were provided by the Institute of Virology and processed at the Institute for Experimental Virology, Twincore - Centre for Experimental and Clinical Infection Research.

Linearized acrylamide (Ambion, Thermo Fisher Scientific; 35 μg/ml final concentration) was added to the sample and total RNA was extracted from 140 μl of aspirate according to the manufacturer’s description (QiaAmp Viral RNA Mini Kit, Qiagen, Hilden, Germany). cDNA synthesis was performed using the Superscript III kit from Invitrogen (Invitrogen, Darmstadt, Germany) and random hexamer primers. Next, a nested PCR was performed first amplifying the RSV-G and F protein coding region and in a second round amplifying the G protein gene. PCR products were sent for Sanger sequencing (GATC, Konstanz, Germany) and the sequences were analyzed using MEGA software and the Highlighter analysis tool [23].

### Ethical approval

We obtained ethical approval for this study from the ethics committee of the Hannover Medical School.

## Results

### Outbreak Setting

The outbreak occurred in the Clinic for Pediatric Hematology and Oncology which is a tertiary referral center for children from 0-18 years with hematologic and solid neoplasia. The affected ward harbors 5 single- and 5 two-bed rooms. Each single room has an anteroom and a high-efficiency particulate air filtration with the air flow directed to the hallway. The same floor houses the outpatient clinic and a recreation room for the hemato-oncological pediatric patients. Exchange from patients between the ward and the outpatient clinic occurs regularly. Moreover two recreation rooms for social activities are part of the ward. Most patients receive antineoplastic therapy during their stay. A substantial number of patients have a neutrophil count below 500 cells / μl. The ward is serviced by permanent health care workers (HCWs) and house-keeping staff. External personnel (such as medical consultants or physiotherapists) enter the ward when necessary. Autologous and allogenic HSCT are performed on a separate ward with 6 single rooms. Parents are allowed to stay overnight with their children.

### Standard infection control measures on the ward

Patients with a positive RSV test are electronically marked in the hospital alert system. Besides single room accommodation, contact and droplet precautions (surgical mask, gown, gloves) are used at any time by visitors and HCWs when entering the room of a RSV positive patient. Affected patients are encouraged to stay in their room and are trained in hygienic hand washing to minimize spread via contact. When leaving the room becomes necessary (e.g. for examination), patients wear a surgical mask. These measures also apply for patients with typical respiratory symptoms (e.g. cough or sneezing) before a pathogen is identified. Measures are usually suspended when there are two negative RSV PCR-based test results at a minimum 2-day interval and therefore patients are not anymore considered infectious. HCWs with symptoms of URTI are suspended from direct patient care and wear a surgical mask while on the ward. Visitors with symptoms of acute RTI are not permitted to enter the ward. Strict hand hygiene following WHO guidelines is implemented. Targeted RSV diagnostics are performed in case of suspected viral RTI. Positive testing for respiratory viruses is regularly reported for epidemiologic and infection control reasons to the infection control staff.

### Cases

**Fig. 1:**
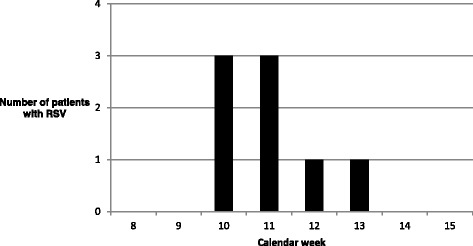
Epidemic curve for the RSV outbreak

**Fig. 2:**
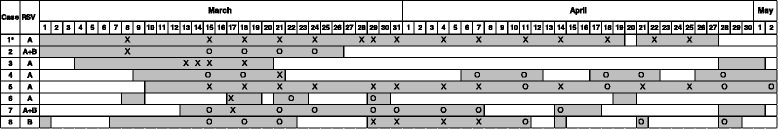
Outbreak timeline. Grey bars indicate the patient’s stay on the ward. ‘X’ indicates positive routine diagnostic RSV testing (DFA, PCR, POCT). ‘O’ indicates a negative routine diagnostic RSV testing. *Case 1 was readmitted on May 6th and had another positive testing on May 9th (not shown)

In March a total of 8 patients (cases 1 to 8) were tested RSV-positive in respiratory samples. Patient characteristics are shown in Table 1. 6 patients fulfilled criteria for nosocomial acquisition. The remaining 2 patients had a possible nosocomial acquisition, as they had been on the ward within 8 days prior to admission. Epidemic curve and an outbreak timeline with the diagnostic results can be seen in Figs 1 and 2.

**Table 1:** Patients’ characteristics

Case	Nosocomial (Yes/No)	Age^a^ (years)	Sex (M/F)	Underlying Disease	RSV infection	WBC^b^ (per microliter)	RSV Treatment, Duration (days)	Additional antibiotic treatment	Oxygen (Yes/No)	RSV-related outcome	Virus shedding (days)
1	yes	16	M	Acute myeloid leukemia (recurrent)	LRTI	0	oral Ribavirin, 64	Yes	Yes	Remission	At least 63
2	yes	15	M	Severe aplastic anemia	LRTI	800	oral Ribavirin, 10	Yes	Yes	Remission	7
3	Yes	16	M	Acute lymphoid leukemia	URTI	1200	-	Yes	Yes (at night)	Remission	At least 6
4^c^	Yes	9	M	Post-transplant Burkitt's leukemia	URTI	300	-	Yes	Yes	Remission	17^d^
5	Yes	1	F	Neuroblastoma	URTI	9500	intravenous Ribavirin, 8	Yes	Yes	Remission	44
6	Possible	3	F	Ewing Sarcoma	URTI	500	-	Yes	Yes	Remission	5
7	Yes	10	F	Acute lymphoid leukemia	URTI	700	-	Yes	No	Remission	4
8	Possible	14	F	Acute lymphoid leukemia	URTI	290	oral Ribavirin, 8	Yes	No	Remission	13

^a^At time of virus detection

^b^At time (+/- 2 days) of virus detection

^c^Onset of disease after discharge (treatment in home town hospital)

^d^There had been no in-house tests between first positive and first negative testing

Case 1 was neutropenic and developed severe LRTI with a RSV positive bronchoalveolar lavage and a requirement for oxygen. Case 2 also suffered from LRTI, which was less severe. Cases 3 to 8 had respiratory symptoms of an URTI such as cough, sneeze and a positive RSV testing in secretions from the upper respiratory tract. Case 6 was co-infected with influenza A virus .

4 patients received oral Ribavirin therapy (case 1, 2, 5, 8) and 5 patients (case 1 to 5) temporarily required supportive oxygen administration via nasal cannula. IVIG or Palivizumab was not administered. No direct RSV-associated mortality was observed. In addition, all patients were empirically treated by antibiotics presuming bacterial co- respectively superinfection according to in-house standards (Table 1).

Viral persistence (viral shedding) is defined as the time period from first positive diagnostic test to sustained negativity. Duration of viral persistence was minimally 4 days (case 7) and maximally at least 63 days (case 1) – see also Table 1.

### Outbreak control measures

Active outbreak management was started after detection of 3 new RSV infected patients in calendar week 10 (see Fig. 1). An outbreak control team consisting of the infection control unit and the physicians in charge was established. The head of the Clinic for Pediatric Hematology and Oncology, the medical director of our institution, and the public health authority were informed. In addition to the existing standard infection control measures described above, interventional measures were introduced. All HCWs (including permanent staff members and external personnel), visitors and outpatients were required to wear a surgical mask at any time (patient care and non-patient care activities) when on the ward and in the outpatient clinic (preemptive barrier precaution). Moreover, roommates of patients tested positive for RSV in their clinical course were moved to single rooms for 8 days (typical maximum incubation period; so called quarantine). They were repeatedly tested for RSV using PCR. All newly admitted patients were tested for RSV (admission screening). Twice weekly PCR RSV prevalence screening for *all* patients on ward was established (prevalence screening). If possible, elective patient admissions were delayed to reduce patient-to-nurse ratio. Moreover, only parents were allowed as visitors. All social activities for the patients and relatives were suspended. During outbreak, all two bed-rooms were occupied by one patient only, meaning single room isolation for *all* patients on the ward. Repeated training sessions for staff were provided by the infection control team. They addressed RSV transmission pathways and underlined the importance of droplet precautions (e.g. masks and cough etiquette for HCWs and visitors) as well as hand hygiene.

Intervention measures were fully implemented on 22^nd^ of March. The last nosocomial case occurred on 29^th^ of March (case 8). However, the patient had been discharged from the ward on 22^nd^ of March, and readmitted on 29^th^ of March (Fig. 2). Thus, after intervention measures had been in place no further nosocomial RSV cases occurred. At the beginning of May the last positive patients were discharged respectively sustainably tested negative and all outbreak control measures were suspended.

### Molecular analysis

**Fig. 3:**
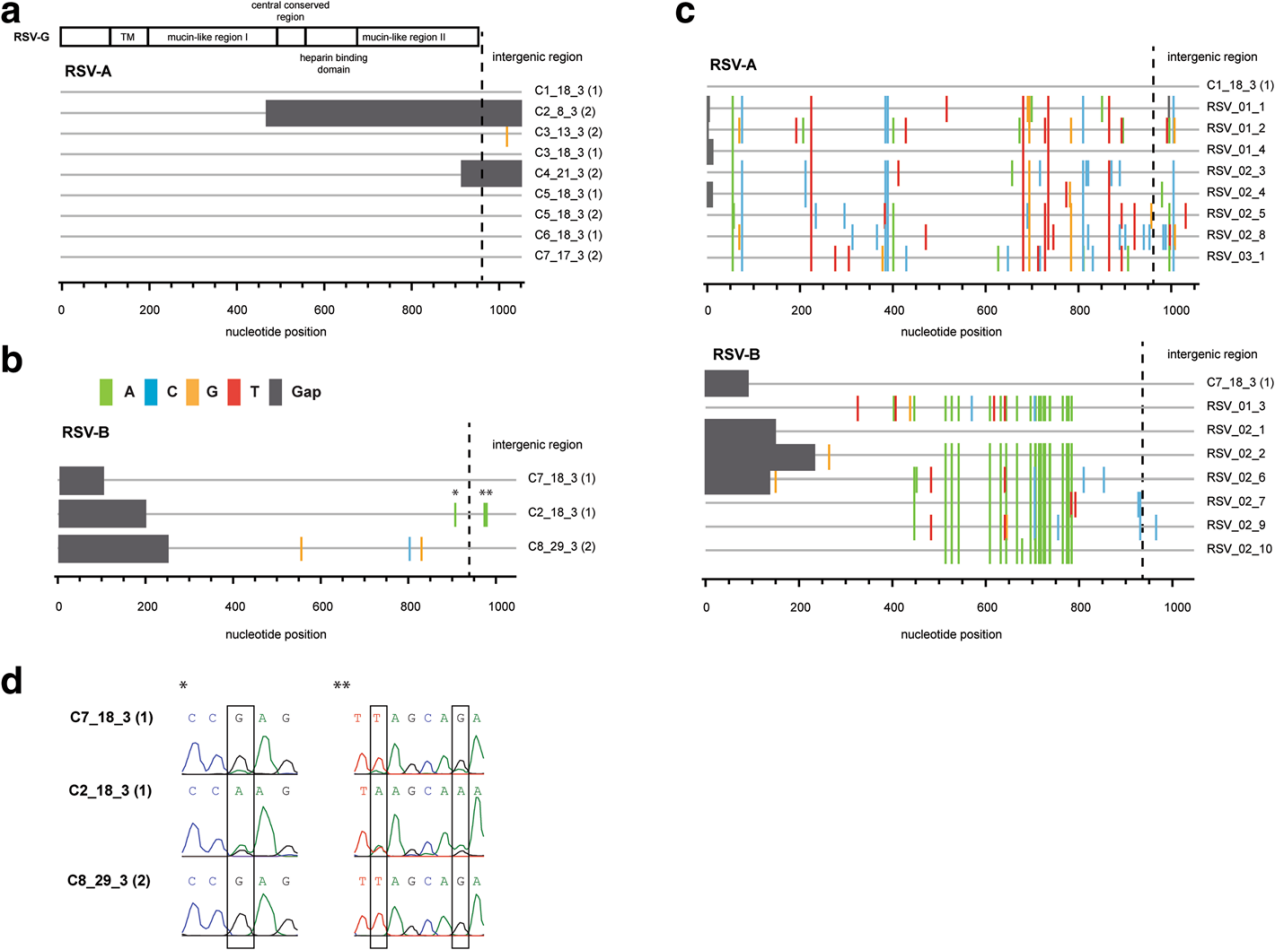
Highlighter plot depicting nucleotide mismatches comparing the sequence of the strain obtained from patient C1 to all other RSV A strains, and the sequence of the strain obtained from patient C7 to all other RSV B strains. **a** RSV A and (**b**) RSV B glycoprotein sequences were aligned using MEGA and depicted as highlighter plot using the highlighter analysis tool [23]. Nucleotide exchanges compared to a reference sequence (C1_18_3 (1) for RSV A and C7_18_3 (1) for RSV B) are depicted in color. Absence of sequence information is depicted as grey bar. A schematic of the RSV G protein with the different domains is depicted on top [modified from [39]]. (1) indicates samples taken exclusively for strain typing at the 18^th^ of March and (2) indicates samples collected for routine viral diagnostics. **c** RSV-G sequence alignment from other pediatric, non-outbreak patients compared to the references C1 and C7, respectively. **d** Sequencing chromatograms for RSV B cases. The depicted area is highlighted by * and ** in Figure 3B. Overlying sequence information from different quasispecies detected in the samples are highlighted in a box

For phylogenetic analysis of the RSV genome we focused on the viral glycoprotein G of RSV as this gene is highly variable and shows the highest sequence variance between the RSV subgroups A and B (53% amino acid sequence identity [24]). From the total of 8 patients, five of them were infected with a RSV A strain (Case 1, 3, 4, 5, 6), one was tested positive for RSV B (case 8) and two patients were infected with RSV A and B (case 2 and 7) dependent on the time point of sampling. Despite the coexistence of genetically definite genotypic strains with many nucleotide exchanges especially in the C-terminal variable region of RSV-G [25–27], we detected the very same nucleotide sequence for the coding region of the RSV-G protein for all patients infected with RSV A (Fig. 3a). Only one single nucleotide exchange was detected in the intergenic region of the virus infecting patient C3 and only at one time point of sampling (Fig. 3a; C3_13_3). In the specimen taken 5 days later (C3_18_3), this variation was no longer detectable. Among the three patients infected with RSV B, we observed 4 nucleotide differences in the coding region of the G protein (Fig. 3b). Two additional variations were observed in the intergenic region of the G gene (Fig. 3b). The RSV B viruses infecting patients C2 and C7 were almost identical with merely three nucleotide differences between them. Notably, the chromatograms of the Sanger sequencing from these patients revealed sequence ambiguity at exactly these three positions: between G/A at position 907, between T/A at position 974 and G/A at residue 979. While in case C7, residues G, T and G were dominant, in patient C2, residues A, A, A predominated. This result suggested that these two patients were infected with an essentially identical viral quasispecies and that merely the relative number of viruses with G, T, G residues compared to viruses with A, A, A nucleotides at these positions varied between these two patients. In contrast, patient C8 carried a RSV B virus with an unambiguous sequence at these three positions G, T, G (Fig. 3d). Moreover, it displayed three additional polymorphisms in the G protein coding region relative to the virus infecting patients C2 and C7, thus supporting the conclusion that this patient was infected with a different RSV B virus. Noticeably, case 2 was tested RSV negative in two samples taken and processed for routine diagnostic on March 15^th^ and 18^th^. RSV strain sequences available from other, non-outbreak pediatric patients (same season as the outbreak) were used for comparison. These strains show a predominantly polyclonal pattern for RSV A (Fig. 3c). Interestingly, one patient (RSV_02_1) was infected with the very same RSV B strain as cases 2 and 7 (Fig. 3c, lower part), whereas there were clear sequence differences for the other RSV B strains isolated from non-outbreak pediatric patients.

## Discussion

For our Clinic of Pediatric Hematology and Oncology this was the first actively managed RSV-outbreak. In the previous two winter seasons in total only 3 RSV-positive patients were detected on the affected ward.

We studied the epidemiologic and molecular background of this outbreak.

Considering bed and room occupancy on the ward during the outbreak, direct patient to patient transmission (e.g. via droplets or contaminated surfaces) in cases 1 and 2 as well as 3 and 4 seemed epidemiologically possible as each pair was accommodated in the same room before samples were tested positive for RSV. Cases 5 and 7 acquired RSV at day 6 and 5 of their stay on the ward, respectively, suggesting nosocomial RSV acquisition. These two patients did not share rooms with other infected patients but were on ward during the outbreak. Cases 6 and 8 were tested positive for RSV on admission. Nosocomial acquisition was considered possible, as case 6 and 8 had been discharged 8 and 7 days, respectively, from the affected ward prior to re-admission. During this previous stay patients with symptoms of RTI and a positive RSV test were already on the ward. Nonetheless, community-onset still was an option for case 6 and 8.

Based on this epidemiologic background, our main hypothesis was that direct and indirect patient to patient transmission (the latter for example via the HCWs’ hands) caused the outbreak. However, at this point transmission by an infected visitor or HCWs acting as a point source could not be excluded. Moreover, taking all epidemiological data into account, a random introduction of several different community-acquired strains seemed unlikely to us. We suspected ongoing transmission of a single RSV variant and sequencing was used retrospectively to test this hypothesis (see below).

The standard, pre-outbreak infection control measures regarding RSV were mainly in line with previously made recommendations for hospitalized patients with hemato-oncologic disease[19, 28]. The additionally implemented measures, in particular single room accommodation for contact patients (quarantine), suspension of all social activities, and surgical masks for all HCWs and visitors at any time, addressed the postulated RSV transmission pathways during this outbreak. These postulated pathways were direct patient to patient transmission (e.g. roommate to roommate), but also transmission via HCWs and visitors.

Direct patient to patient transmission as the most probable route of infection has been shown by Lehners et al. in a large RSV outbreak in a German hematology and transplant unit [11]. Jensen et al. described direct patient to patient transmission, mixed with introduction of strains from outside, in an outbreak affecting immunocompromised adults [29]. We therefore focused on patient to patient transmission early during the outbreak by strict isolation precautions for RSV infected patients and contacts. Isolation for infected patients was also a key measure in a multimodal control bundle described by Inkster et al. [15]. Contact patients were isolated for 8 days and repeatedly tested in order to disrupt infection chains as described in literature [11]. This so called quarantine concerned 2 patients in our outbreak. One of them (case 4) was eventually tested RSV-positive at day 8 of quarantine while being negative at day 2 and 5. This underlines the value of the measure. Finally, we re-emphasized in training sessions the need for preemptive isolation of patients with respiratory symptoms. As all these measures required more isolation capacity on the ward, we restricted elective admissions and located all patients in single rooms.

As another measure we reduced direct patient to patient contacts on the ward by suspending community events, as active social behavior can be a risk factor for nosocomial RSV acquisition [30]. Even so this noticeably restricted the social life for the patients and their families during the outbreak, we enforced this measure. We further restricted social contacts by temporally limiting visits of infants to the ward, as (especially young) infants are known to be the main reservoir for RSV and as our outbreak was approximately concurrent (slightly delayed) to the RSV community peak. Only parents were allowed to the ward, which is in line with an intervention done by Kelley et al. [12]. A restrictive visiting policy is as well described by Singh et al. in a pediatric RSV outbreak [14].

The use of surgical masks for everyone on the ward is an important measure to prevent droplet associated nosocomial RSV transmissions. This is even more rational as RSV may be transmitted via symptomless or oligosymptomatic persons (e.g. HCWs or visitors) and the infectious period can in fact already begin 1 to 2 days before actual onset of symptoms. A literature review by French et al. concluded that personal protective equipment might be advantageous for reducing nosocomial RSV transmission [31]. Kelly et al. showed that five HCWs showing only mild symptoms were involved in a RSV outbreak on an adult stem cell transplant unit [12]. This underlines the necessity that HCWs with respiratory symptoms should not participate in direct patient care activities, at least in a high risk patient care setting. We re-emphasized this issue in training sessions for the HCWs. Although staff screening is described in literature [15], we were able to terminate this outbreak without staff screening. A cohort of HCWs to take care of solely RSV-positive patients as reported before [9] had also not been established but would have been another option in case of an ongoing outbreak.

Temporal survival of respiratory viruses in general [32] and specifically RSV [33] on inanimate surfaces is described, thus contact transmission via the hands of staff was conceivable for nosocomial acquisition. This is especially of importance as cough etiquette and compliance to basic hygienic principles may be reduced for obvious reasons in pediatric patients, so a higher environmental RSV burden is probable. Nonetheless we did not implement changes in the well established cleaning and disinfection procedures on the ward.

We detected prolonged RSV persistence (virus shedding), which has been reported in patients with hematological disorders [34]. This finding needs to be considered for efficient outbreak control and favors the practice of repeated testing in immunocompromised patients as we did. Likewise, this is important as pediatric hemato-oncologic patients are often readmitted several times for cancer treatment cycles or fever in neutropenia. When symptoms are no longer present or mild but viruses are still being shed, RSV may be re-introduced to the ward. Thus, for termination of isolation precautions during the outbreak, we required negative results as reported before [35]. In fact, two subsequent negative results at a minimum 2-day interval were necessary. The usefulness of this requirement is supported by the longitudinal course of the samples from patient 5 which were obtained in April and May. This patient produced positive specimens on two occasions, after one specimen had been tested negative (see Fig. 2).

Active RSV-surveillance by screening on admission and twice weekly for all patients on the ward insured rapid detection of RSV-positive patients. This is in line with successful infection control measures reported in literature [9, 12]. We presume that a prophylactic admission and prevalence RSV screening for all patients in the winter season might be helpful as a preventive measure in high risk populations. Therefore, one consequence of this outbreak was the implementation of an active RSV surveillance (admission and prevalence screening once weekly) in our Clinic for Pediatric Hematology and Oncology during the RSV season. The beginning and ending of this seasonal screening period is determined by in-house and regional/national RSV epidemiology [36]. Moreover, pre-RSV-season audits involving clinicians, infection control staff and the Institute of Virology take place to ensure timely beginning of screening procedures and adherence to the existing infection control practices.

Molecular characterization of RSV strains, for instance by whole genome sequencing [37] or characterization of RSV G-Protein [16, 38], has been used to investigate nosocomial RSV outbreaks. We were able to collect and examine selected outbreak strains by G-Protein gene sequencing. We found that cases 1 to 7 were infected with an RSV A virus with identical G protein coding region. In case of patient C3 one nucleotide difference in the intergenic region of the G gene was observed in one of two samples collected five days apart (Fig. 3a. It is possible that this change was due to natural drift of the infecting virus over time or that this polymorphism is indicative of the presence of two slightly different viruses replicating in parallel and dominating on the one and the other day of sampling, respectively.

Moreover we found that case 8 had a RSV-B infection and that cases 2 and 7 were co-infected by RSV A and RSV B viruses. While sequence analysis of the earlier samples of case 2 and 7 revealed infection by the RSV A virus, the sequence analysis of the later specimen showed infection by an RSV B virus. With the available specimen, we were unable to distinguish if these two patients had a prolonged co-infection between these viruses or if they were sequentially infected by RSV A and RSV B. These findings became available only after the outbreak ended, as routine virological testing during the outbreak did not include molecular differentiation of RSV A and B. In retrospect, these results indicated the decision not to cohort RSV-patients during the outbreak, as we probably might have cohorted RSV-patients with different subtypes. Detailed sequencing analysis suggests that cases 2 and 7 were infected by an almost identical RSV B virus population. We observed three nucleotide differences between these viruses; however, nucleotides of the viruses at these three positions were ambiguous in both cases (G,T,G versus A,A,A residues). Thus, both patients were likely infected by a highly similar RSV B quasispecies which was characterized by two different nucleotide signatures varying in abundance between patients. In contrast, the RSV B virus infecting patient C8 differed in two key criteria. First, it did not show any sequence ambiguity at the three above mentioned residues that was characteristic for the RSV B virus population observed in patients C2 and C7. Second, it displayed three additional polymorphisms in the coding region of the G protein. Taken together, this suggests that patient C8 was infected by another RSV B virus and that there was no transmission from patients C2 and C7 to patient C8.

Outbreak strains of the subtype RSV A were highly similar and different from polyclonal strains from other non-outbreak pediatric patients (Fig. 3c). We therefore conclude that a single RSV A strain was introduced to the ward and then spread within the ward. Interestingly, the RSV B isolate C7_18_3 (1) was identical to the community strain RSV_02_1, however further epidemiologic and clinical information are not accessible for the non-outbreak patient. Taken together, the nucleotide analysis suggests independent introductions of at least 2 different RSV B strains into the ward affecting patient C2, C7 and C8, and transmission of one RSV A strain on the ward between patients C1 to C7.

Looking exclusively at the molecular analysis, it is not possible to disclose the exact transmission pathway of RSV A. RSV A might have been introduced to the ward by an infected patient (index patient) on the ward (maybe case 1) and was then successively transmitted from patient to patient. Alternatively, a point source, such as a RSV-positive HCW, may have caused the outbreak. However, in correlation with the epidemiologic observations such as overlapping patient stays on the ward, stay of case 1 and 2, and case 3 and 4 in a double room, and social activity on the ward in the initial phase of the outbreak, we consider a direct and indirect patient to patient transmission most likely.

## Conclusions

RSV poses a significant infectious threat to pediatric patients with an underlying oncologic disease. This outbreak and other outbreaks reported in literature demonstrate the potential of RSV to spread in a hospital. We strictly enforced our existing infection control practices and implemented temporally additional measures to terminate the outbreak. According to our experiences an outbreak control bundle for RSV should include (preemptive) barrier precautions (especially masks), prevalence and admission screenings for *all* patients, and strict isolation procedures for infected patients and contact patients. Quarantine for contacts should at least be for 8 days, the usual maximal incubation period of RSV. In pediatric settings the restriction of visitors (especially siblings) and social activities on the ward can be helpful to prevent transmission and RSV introduction from outside, but definitely limits social life quality. As shown in other outbreaks with viral and bacterial pathogens restriction of admissions still is a very effective measure as it enables single room accommodation for all or the majority of the patients. Moreover, a decrease of the patient to nurse ratio makes transmission more unlikely. In our case the molecular analysis was very helpful to verify the true outbreak character of the RSV cluster and revealed ongoing transmission of an unique RSV A strain on the ward, and an probable independent introduction of different RSV B strains into the ward.

## Abbreviations

RSV: Respiratory syncytial virus
URTI: Upper respiratory tract infection
LRTI: Lower respiratory tract infection
HSCT: Hematopoietic stem cell transplantation
RTI: Respiratory tract infection
IVIG: Intravenous immunoglobulins
DFA: Direct fluorescent antibody staining
FITC: Fluorescein isothiocyanate
POCT: Point-of-care-testing
HCWs: Health care workers
